# Structure, Function, and Evolution of the *Thiomonas* spp. Genome

**DOI:** 10.1371/journal.pgen.1000859

**Published:** 2010-02-26

**Authors:** Florence Arsène-Ploetze, Sandrine Koechler, Marie Marchal, Jean-Yves Coppée, Michael Chandler, Violaine Bonnefoy, Céline Brochier-Armanet, Mohamed Barakat, Valérie Barbe, Fabienne Battaglia-Brunet, Odile Bruneel, Christopher G. Bryan, Jessica Cleiss-Arnold, Stéphane Cruveiller, Mathieu Erhardt, Audrey Heinrich-Salmeron, Florence Hommais, Catherine Joulian, Evelyne Krin, Aurélie Lieutaud, Didier Lièvremont, Caroline Michel, Daniel Muller, Philippe Ortet, Caroline Proux, Patricia Siguier, David Roche, Zoé Rouy, Grégory Salvignol, Djamila Slyemi, Emmanuel Talla, Stéphanie Weiss, Jean Weissenbach, Claudine Médigue, Philippe N. Bertin

**Affiliations:** 1 Génétique Moléculaire, Génomique et Microbiologie, UMR7156 CNRS and Université de Strasbourg, Strasbourg, France; 2 Genopole, Plate-forme puces à ADN, Institut Pasteur, Paris, France; 3 Laboratoire de Microbiologie et Génétique Moléculaire, UMR5100, Toulouse, France; 4 Laboratoire de Chimie Bactérienne, UPR9043 CNRS, Institut de Microbiologie de la Méditerranée, Marseille, France; 5 Institut de Biologie Environnementale et de Biotechnologie, CEA-CNRS-Université Aix-Marseille II, Saint-Paul-lez-Durance, France; 6 Institut de Génomique, CEA-DSV, Génoscope, Evry, France; 7 Environnement et Procédés, Ecotechnologie, BRGM, Orléans, France; 8 Laboratoire Hydrosciences Montpellier, UMR 5569 CNRS, IRD and Universités Montpellier I and II, Montpellier, France; 9 Génomique Métabolique, Laboratoire de Génomique Comparative, CNRS UMR8030, Evry, France; 10 Institut de Biologie Moléculaire des Plantes, CNRS, Université de Strasbourg, Strasbourg, France; 11 Unité Microbiologie, Adaptation, Pathogénie, CNRS-INSA-UCB UMR 5240, Université Lyon 1, Villeurbanne, France; 12 Génétique des Génomes Bactériens, URA2171, Institut Pasteur, Paris, France; University of Arizona, United States of America

## Abstract

Bacteria of the *Thiomonas* genus are ubiquitous in extreme environments, such as arsenic-rich acid mine drainage (AMD). The genome of one of these strains, *Thiomonas* sp. 3As, was sequenced, annotated, and examined, revealing specific adaptations allowing this bacterium to survive and grow in its highly toxic environment. In order to explore genomic diversity as well as genetic evolution in *Thiomonas* spp., a comparative genomic hybridization (CGH) approach was used on eight different strains of the *Thiomonas* genus, including five strains of the same species. Our results suggest that the *Thiomonas* genome has evolved through the gain or loss of genomic islands and that this evolution is influenced by the specific environmental conditions in which the strains live.

## Author Summary

Recent advances in the field of arsenic microbial metabolism have revealed that bacteria colonize a large panel of highly contaminated environments. Belonging to the order of Burkholderiales, *Thiomonas* strains are ubiquitous in arsenic-contaminated environments. The genome of one of them, i.e. *Thiomonas* sp. 3As, was deciphered and compared to the genome of several other *Thiomonas* strains. We found that their flexible gene pool evolved to allow both the surviving and growth in their peculiar environment. In particular, the acquisition by strains of the same species of different genomic islands conferred heavy metal resistance and metabolic idiosyncrasies. Our comparative genomic analyses suggest that the natural environment influences the genomic evolution of these bacteria. Importantly, these results highlight the genomic variability that may exist inside a taxonomic group, enlarging the concept of bacterial species.

## Introduction

In environments such as those impacted by acid mine drainage (AMD), high toxic element concentrations, low levels of organic matter and low pH make growth conditions extreme. AMD is generally characterized by elevated sulfate, iron and other metal concentrations, in particular, inorganic forms of arsenic such as arsenite (As(III)) and arsenate (As(V)) [1],[2]. While these waters are toxic to the majority of prokaryotic and eukaryotic organisms, a few Bacteria and Archaea are not only resistant to but also able to metabolize some of the toxic compounds present [1]. Members of the *Thiomonas* genus are frequently found in AMD and AMD-impacted environments, as *Thiomonas* sp. 3As and “*Thiomonas arsenivorans*” [2]–[6]. These *Betaproteobacteria* have been defined as facultative chemolithoautotrophs, which grow optimally in mixotrophic media containing reduced inorganic sulfur compounds (RISCs) and organic supplements. Some strains are capable of autotrophic growth and others are capable of organotrophic growth in the absence of any inorganic energy source [5],[7],[8]. Recently described species and isolates include *“Tm. arsenivorans”* [9], *Tm. delicata* [10], *Thiomonas* sp. 3As [5] and Ynys1 [11]. *Thiomonas* sp. 3As as well as other recently isolated strains from AMD draining the Carnoulès mine site (southeastern France) containing a high arsenic concentration (up to 350 mg L^−1^) [3],[12], present interesting physiological and metabolic capacities, in particular an ability to oxidize As(III).

Over the past few years an increasing number of genomes has been sequenced, revealing that bacterial species harbor a core genome containing essential genes and a dispensable genome carrying accessory genes [13]. Some of these accessory genes are found within genomic islands (GEIs) [14] and have been acquired by horizontal gene transfer (HGT). These GEIs are discrete DNA segments (from 10 to 200 kbp) characterized by nucleotide statistics (G+C content or codon usage) that differ from the rest of the genome, and are often inserted in tRNA or tRNA-like genes. Their boundaries are frequently determined by 16–20 bp (up to 130 bp) perfect or almost perfect direct repeats (DRs). These regions often harbor functional or cryptic genes encoding integrases or factors involved in plasmid conjugation or related to phages. GEIs encompass other categories of elements such as integrative and conjugative elements (ICE), conjugative transposons and cryptic or defective prophages. Such GEIs are self-mobile and play an important role in genome plasticity [14]. In almost all cases, GEIs have been detected *in silico*, by the comparison of closely related strains. Nevertheless, the role of GEIs in genome plasticity has also been experimentally demonstrated in several pathogenic bacteria such as *Staphylococcus aureus* or *Yersinia pseudotuberculosis* [15],[16] or in *Pseudomonas* sp. strain B13 isolated from a sewage treatment plant [17].

Deciphering dispensable genomes has revealed that the loss or gain of genomic islands may be important for bacterial evolution [18]. Indeed, these analyses allow the determination of the genome sequence, called pan-genome or supragenome, not just of individual bacteria, but also of entire species, genera or even bacterial kingdom [19],[20]. These data result in debates on taxonomic methods used to define the bacterial species [21],[22], e.g. pathogens such as *Streptococcus agalactiae* [21],[23] or environmental bacteria such as *Prochlorococcus* [24],[25] or *Agrobacterium* [26]. However, beyond these well-known and cultivable microorganisms, the diversity of bacteria, in particular those found in extreme environments, has hitherto been comparatively poorly studied. Genome analysis of such extremophiles may yet reveal interesting capacities since these bacteria may express unexpected and unusual enzymes [27]. Since the role of GEIs in extremophiles has not been yet well explored, little is known about their evolution.

In the present study, the genome of *Thiomonas* sp. 3As was sequenced and analyzed. It was next compared to the genome of other *Thiomonas* strains, either of the same species or of other species of the same genus. This genome exploration revealed that *Thiomonas* sp. 3As evolved to survive and grow in its particular extreme environment, probably through the acquisition of GEIs.

## Results

### General Features of the *Thiomonas* sp. 3As Genome

The genome of *Thiomonas* sp. 3As comprises a 3.7 Mbp circular chromosome and a 46.8 kbp plasmid (Table 1). The single circular chromosome contains 3,632 coding sequences (CDSs) (Table 1, Figure S1A). The mean G+C content of the *Thiomonas* sp. 3As genome is 63.8%. However, the distribution along the genome revealed several regions with a G+C content clearly divergent from this mean value (Figure S1). This suggests that several genomic regions are of exogenous origin. Indeed, 196 genes having mobile and extrachromosomal element functions were identified in the genome, among which a total of 91 ISs (Figure S1A, Table S1) representing 2.5% of total CDS. None of these ISs were found as part of composite transposons, while several were identified as neighbors of phage-like site-specific recombinases.

**Table 1:** General genome and plasmid features.

Molecule	Category	Feature	Value
Plasmid	General characteristics	Size (bp)	46,756
		GC content (%)	60.49
		Coding density (%)	88.19
		Predicted CDSs	68
	Proteins with predicted function	Percent of total CDSs	48.52
		Secretion (%)	11.76
		Partitioning	2.94
		Replication, recombination	8.82
		Inorganic ion transport and metabolism (Hg)	2.94
	Proteins without predicted function	Conserved hypothetical proteins (%)	13.24
		No homology to any previously reported sequences (%)	38.24
		Percent of total CDSs	51.48
Genome	General characteristics	Size (bp)	3,738,778
		GC content (%)	63.8
		Coding density (%)	90.01
		16S-23S-5S rRNA operons	1
		tRNAs	43
		Predicted CDSs	3,632
	Proteins with predicted function	Percent of total CDSs	74.2
		Heavy metal resistance (%)	1.6
		Related to arsenic metabolism/transport (%)	0.6
	Proteins without predicted function	Conserved hypothetical proteins (%)	13.54
		Hypothetical proteins (%)	11.95
		Percent of total CDSs	25.8
	Mobile and extrachromosomal element functions	Percent of total CDSs	5.4
		Transposases (nb CDSs)	101
		Phage related (nb CDSs)	61
	Repeated regions (%)		7.62

The plasmid, pTHI, contains 68 predicted CDS. 21 genes were found in synteny with genes carried by the *R. eutropha* JMP134 plasmid pJP4, and among them, *par*/*trf*/*pem* genes necessary for plasmid partitioning, stability and replication (Figure S1B). These observations suggest that pTHI, as JMP134, belongs to the IncP-1β group [28]. pTHI contains 13 of the 14 genes involved in conjugation (*vir* and *tra* genes) and genes that could fulfill the function of the missing components were found on the chromosome. Therefore, *Thiomonas* sp. 3As may be able to express a complete Type IV secretory system (T4SS) of the Vir/Tra type required for pTHI conjugal transfer. IncP-1β members are known to carry multi-resistance determinants and degradative cassettes [28], and plasmid pTHI indeed carries a Tn*3*-related transposon. This transposon contains part of a mercury resistance operon found in many other Gram negative bacterial transposon such as Tn*21*, Tn*501* and Tn*5053* [29].

### Carbon and Energy Metabolism

**Figure 1:**
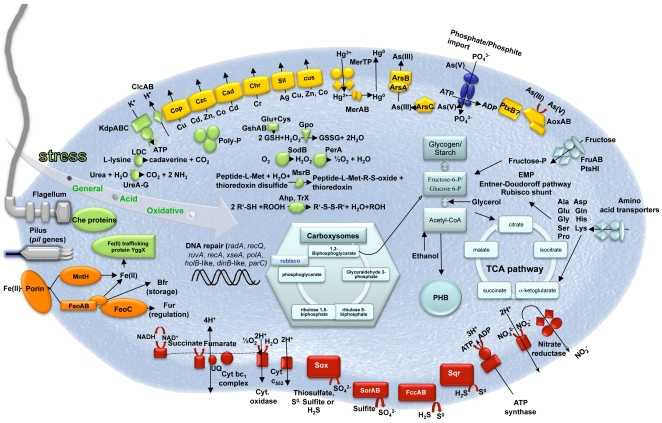
Schematic representation of the major metabolic pathways in *Thiomonas* sp. 3As. These metabolic pathways were ascertained using genome and physiological data ([5], this study). Light blue, carbon metabolism; Red, oxidative phosphorylation; Green, stress response; Yellow, heavy metal resistance; Orange, iron metabolism; Dark blue, phosphate import (*pst* genes).

*Thiomonas* sp. 3As is able to use organic compounds as a carbon source or electron donor [5],[8]. However, under certain conditions this bacterium may also be able to grow autotrophically [5]. A complete set of *cbb/rbc*/*cso* genes involved in carbon fixation *via* the Calvin cycle, and genes involved in glycogen, starch and polyhydroxybutyrate (PHB) biosynthesis pathways were identified (Figure 1 and [5]). Fructose, glucose, several amino acid, C4-dicarboxylates, propionate, acetate, lactate, formate, ethanol and glycerol are potential carbon sources or electron donors, since genes involved in their import or degradation *via* the glycolysis, the Entner-Doudoroff, the TriCarboxylic Acid (TCA) or the “rubisco shunt” pathways are present in the genome. The presence of all genes involved in the oxidative phosphorylation pathway (Figure 1) suggests that *Thiomonas* sp. 3As has a respiratory metabolism. Moreover, since several genes coding for terminal oxidases (i.e. *cbb*_3_, *bd* or *aa*_3_) were found, this respiratory metabolism may occur over a wide range of oxygen tensions. Finally, the presence of a nitrate reductase and of several formate dehydrogenases suggests that *Thiomonas* sp. 3As is able to use nitrate anaerobically as an electron acceptor and formate as electron donor. In the absence of carbohydrates, *Thiomonas* sp. 3As is a chemolithotroph and may use reduced inorganic sulfur compounds (RISCs) as an electron donor [5]. The presence of *soxRCDYZAXB*, *dsr*, *sorAB*, *sqr* and *fccAB* genes revealed that *Thiomonas* can oxidize thiosulfate, sulfite, S^0^ or H_2_S to sulfate (Figure 1) [30],[31].

### Adaptive Capacities of *Thiomonas* sp. 3As to Its Extreme Environment

*Thiomonas* sp. is a moderate acidophile. Its optimum pH is 5 but this bacterium can withstand to pH as low as 2.9 (Slyemi, Johnson and Bonnefoy, personal communication). *Thiomonas* sp. 3As pH homeostasis mechanisms may therefore be strictly controlled as previously described [32],[33]. First, genes encoding a potassium-transporting P type ATPase (*kdpABC*) are present in the *Thiomonas* sp. 3As genome. This ATPase could be involved in the generation of a positive internal potential produced by a greater influx of potassium ions than the outward flux of protons. Second, to strengthen the membrane, likely by lowering membrane proton conductance, *Thiomonas* sp. has cyclopropane fatty acids [5]. Accordingly, two putative *cfa* genes encoding cyclopropane fatty acid synthase have been detected. Third, cytoplasmic buffering can be mediated either by amino acid decarboxylation and/or by polyphosphate granules. Genes encoding decarboxylases for lysine (4 CDS), phosphatidyl serine and glycine are present on *Thiomonas* sp. 3As genome. Moreover, urea (formed from arginine by arginase) may be degraded by urease (*ure* genes) or urea carboxylase and allophanate hydrolase. Urease encoding genes are known to be involved in acid tolerance in *Helicobacter pylori* [34]. Protons may be captured during polyphosphate synthesis. Polyphosphate granules have indeed been observed in electron micrographs of thin sections of *Thiomonas* sp. 3As [5]. Genes involved in such mechanisms (*ppk*, *pap*, *ppx*) were found in 3As genome. Fourth, primary and secondary proton efflux transporters were predicted by genome sequence analysis, including four putative Na^+^/H^+^ exchangers and voltage gated channels for chloride involved in the extreme acid resistance response in *E. coli* (*clcAB*) [35]. Finally, the elimination of organic acids can lead to pH homeostasis. Some organic acid degradation pathways have been detected in *Thiomonas* sp. such as an acetyl-CoA synthetase-like. Moreover, formate oxidation was observed (Slyemi, Johnson and Bonnefoy, personal communication) and two formate dehydrogenases are encoded by the *Thiomonas* sp. 3As genome, these enzymes could convert acetate to acetyl-CoA and formate to CO_2_ and hydrogen, respectively.

The Carnoulès AMD contains a high concentration of heavy metals such as zinc or lead. To resist to heavy metals, bacteria usually develop several resistance mechanism including toxic compounds extrusion pumps [36] or biofilm synthesis [37]. Flagella are important for the first steps of biofilm formation and all genes involved in motility, twitching and chemotaxis, were found in its genome. *Thiomonas* sp. 3As is motile but, unlike *H. arsenicoxydans*, this motility was not affected by arsenic concentration (Table S2, [38]). Finally, *Thiomonas* sp. 3As is able to synthesize exopolysaccharides (Table S2), and one *eps* operon involved in their synthesis was identified in the genome, as well as two *mdoDG* clusters involved in glucan synthesis. Several genes conferring resistance to cadmium, zinc, silver, (*cad*, *czc*, and *sil* genes), chromium (*chr* genes), mercury (2 *mer* operons encoding both MerA reductase but no organomercury lyase MerB), copper (*cop* and *cus* genes) and tellurite (transporters THI_0898-0899) are likely involved in *Thiomonas* sp. 3As heavy metal resistance (Figure 1). Arsenic resistance in bacteria is partly due to the expression of *ars* genes, among which, *arsC* encodes an arsenate reductase, *arsA* and *arsB* encode an arsenite efflux pump, *arsR* encodes a transcriptional regulator [39]. *Thiomonas* sp. 3As is resistant to up to 50 mM As(V) and up to 6 mM As(III) (Table S2, [8]). The analysis of the *Thiomonas* sp. 3As genome revealed the presence of two copies of the *ars* operon, an *arsRBC* operon (*ars1*) and an *arsRDABC* operon (*ars2*). *Thiomonas* sp. 3As is able to oxidize As(III) to As(V) [5] and this metabolism involves the arsenite oxidase encoded by *aoxAB* genes [5] (Figure 1). It has been shown that arsenite is imported *via* the aquaglyceroporin GlpF in *E. coli* [40]. However, as in *H. arsenicoxydans* [38], no homologue of GlpF was identified in the *Thiomonas* sp. 3As genome, suggesting that As(III) is imported *via* an unknown component.

As(III) is known to induce DNA damage and oxidative stress [41],[42]. 24 genes involved in such stress responses were identified in the *Thiomonas* sp. 3As genome (Figure 1). Moreover, this genome carries 54 genes involved in DNA repair. However, this strain lacks some genes present in *H. arsenicoxydans*, such as *alkB*, whereas two genes involved in mismatch repair were duplicated. Orthologs of genes that have been shown to be induced in response to arsenic in *H. arsenicoxydans* [38] were found in *Thiomonas* sp. 3As, i.e. *radA*, *recQ*, *ruvA*, *recA*, *xseA*, *polA*, *holB*-like, *dinB*-like and *parC*. The expression of *polA* has been previously shown to be induced in the presence of arsenic [8], suggesting that the *Thiomonas* sp. 3As response to arsenic include the expression of genes involved in DNA repair.

### Comparative Genomic Analyses Allowed the Definition of 19 Genomic Islands (GEIs)

**Figure 2:**
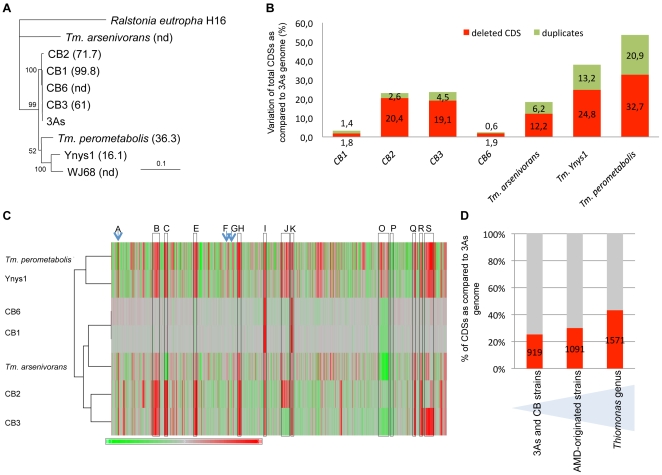
Genomic diversity among *Thiomonas* strains. (A) Phylogenetic dendrogram of the SuperGene construct of both the 16S rRNA and *rpoA* genes of the *Thiomonas* strains used in this study. *Ralstonia eutropha* H16 served as the outgroup. The DNA-DNA hybridization percentage is included in brackets. nd: not determined (CB1 and CB6 were almost identical at the genetic content level. For this reason, DNA-DNA hybridization analyses were performed only with CB1). Numbers at the branches indicate percentage bootstrap support from 500 re-samplings for ML analysis. NJ analyses produced the same branch positions (not shown). The scale bar represents changes per nucleotide; (B) number of variable CDS exhibited by each strain determined by CGH, expressed as a percent of the total number of 3As CDS; (C) hierarchical clustering established based on CGH experiments, represented as a composite view of genome diversity in *Thiomonas* strains compared to 3As. The column represents genes as they are found along 3As genome, starting from the origin of replication (THI0001). Results for each strain are shown in each row. Red color indicates absence or strong divergence leading to reduced hybridization efficiency as compared to the corresponding *Thiomonas* sp. 3As gene (Log_2_(A_635 nm_/A_532 nm_) ≤−1)); Green color indicates a Log_2_(A_635 nm_/A_532 nm_) ≥1, suggesting duplication of this gene. The regions corresponding to ThGEI-A, B, C, E, F, G, H, I, J, K, O, P, Q, R and S are indicated by a blue arrow or a grey rectangle; (D) percent of CDS found in core (in grey) or in dispensable (in red) genome, in 3As and CB strains (1^st^ column), in AMD-originated strains (3As, CB strains and “*Tm. arsenivorans”*, 2^nd^ column), or in all *Thiomonas* strains (3^rd^ column).

Several *Thiomonas* strains called CB1, CB2, CB3 and CB6 were isolated from the same environmental site as *Thiomonas* sp. 3As. The 16S rRNA/*rpoA*-based phylogeny of these isolates (>97% nucleotide identity), as well as DNA-DNA hybridization experiments (Figure 2A), revealed that they represent different strains of the same species. All these strains are able to oxidize As(III) and are resistant to As(III) (Table S2). Nevertheless, subtle physiological differences were observed (Table S2).

**Figure 3:**
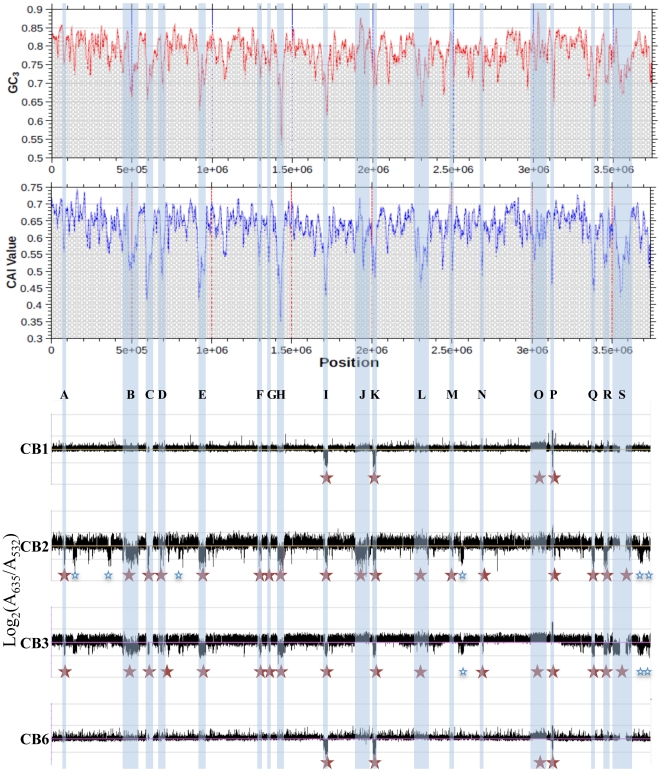
Deleted or duplicated regions in CB strains, obtained by CGH experiments. The codon adaptation index (CAI) and the GC content at the 3^rd^ nucleotide position of each codon (GC_3_) of *Thiomonas* sp. 3As is shown in the upper part. Y-axis displays the log2-ratios (Cy5 (measured at 635 nm)/Cy3 (measured at 532 nm)). Red stars: variable regions found in CB strains and corresponding to GEIs; Blue stars: other variable regions.

The existence of both phylogenetical relationships and physiological differences between these strains prompted us to perform a comparative genome analysis in order to address the evolution of *Thiomonas* strains. Therefore, genome variability was searched for by investigating genetic similarities and diversities among these closely related *Thiomonas* strains, using a Comparative Genomic Hybridization (CGH) approach (Figure 3). These experiments revealed the presence of a flexible CDS (duplicated, absent or highly divergent) pool in CB1, CB6 CB3 and CB2 (Figure 2B, Figure 3, ArrayExpress database, accession number E-MEXP-2260) representing 2.5%, 3.2%, 24.1% and 23.1% of the genome of strain 3As (Figure 2B), respectively. Altogether, these experiments led to the definition of 919 dispensable CDS, i.e. absent or highly divergent in at least one strain, accounting for 25.3% of strain 3As genes (Figure 2D). The remaining conserved CDS (2713 CDS, 74.7% of the genome of strain 3As) represent a common backbone of the “core” genes of this species.

In order to enlarge our comparative analysis, genomic similarities were similarly searched for in other *Thiomonas* species: an arsenite-oxidizing strain, *“Tm. arsenivorans”*, and two closely related strains that are unable to oxidize arsenite, *Tm. perometabolis* and *Thiomonas* sp. Ynys1 (Table S2, Figure 2, ArrayExpress database, accession number E-MEXP-2260). No significant hybridization was observed with oligomers corresponding to the plasmid, suggesting that pTHI is absent in all these strains. 18.4, 37.9 and 53.6% of the 3As CDS were flexible in *“Tm. arsenivorans”*, Ynys1 and *Tm. perometabolis*, respectively. Altogether, 1571 CDS accounting for at least 43.3% of the *Thiomonas* sp. 3As genome were found in the *Thiomonas* genus dispensable genome (Figure 3D). Finally, these CGH experiments revealed that the *Thiomonas* core genome contains 2,061 CDS (56.7% of the *Thiomonas* genome). Interestingly, almost all genes involved in acid resistance described above, were found in this core genome, as for example genes involved in polyphosphate granule synthesis, *cfa* and *kdp* genes, genes encoding ion transporter amino acid decarboxylase, formate dehydrogenase and other hydrogenases. One *ars* operon involved in arsenic resistance, i.e. *ars1*, and almost all genes involved in DNA repair were also conserved in all strains.

Among the flexible pool, 19 regions (ThGEI-A - ThGEI-S) had similarities with GEIs found in other bacterial genomes, suggesting that they were possibly acquired by horizontal gene transfer: (i) an abnormal deviation of the codon adaptation index (CAI) and the GC content at the 3^rd^ nucleotide position of each codon (GC_3_) was observed in these regions as compared to the rest of the genome (Figure 3), (ii) many of their genes formed syntenic blocks that differed from the general synteny observed in the rest of the genome (Table S3), (iii) genes with mobile and extrachromosomal element functions such as those coding for integrases were localized within these regions, (iv) these regions were present at the 3′-end of tRNA or miscRNA genes, and/or (v) the borders of five deletions were verified in CB strains, by PCR and direct repeats (10 to 112 bp-long) bordering these GEIs were found (Table S3).

### Genetic Content of the 19 Genomic Islands Found in *Thiomonas* sp. 3As Genome

Genes found in the 19 *Thiomonas* sp. 3As GEIs and the syntenies they share with genes in other bacteria are shown in Table S3. Interestingly, 70 (76.9%) of the 91 complete and partial ISs identified in the genome were located in genomic islands which represent only 21.5% of the genome (Figure S1A). In addition to the high numbers of ISs found in these GEIs, many hypothetical proteins as well as modification/restriction enzymes were encoded by these regions. In ten GEIs, accessory genes are involved in a particular metabolism such as acetoin, atrazin, benzoate, ethyl tetra-butyl ether (ETBE) hydroxyisobutyrate phenylacetic acid and urea degradation (ThGEI-E, ThGEI-C, ThGEI-S or ThGEI-R), or heavy metal resistance (ThGEI-J, ThGEI-L, ThGEI-O).

Interestingly, several genes found in distinct GEIs shared high amino acid identity (>70%, Figure S2). In addition, 47 genes found in the two regions ThGEI-C and ThGEI-S shared 100% identity. Because of this duplication, a 7 kbp region in ThGEI-S could not be sequenced and this gap may correspond to duplicated genes of ThGEI-C. These observations suggest that genomic rearrangements occurred between several GEIs. Moreover, several islands seem to be composite, since some fragments of such islands are deleted or duplicated in *Thiomonas* strains. Such composite structure may originate from insertion or excision of DNA elements in these GEIs, which involve integrase or excisionase. This hypothesis is strengthened by the observation that 32 integrases were found in almost all GEIs except for ThGEI-B and ThGEI-R. Some of such integrases are similar to phage integrases. In addition, 2 excisionases are present in ThGEI-H and ThGEI-P and such genes were localized in the vicinity of tRNA, an additional phage-like character.

**Figure 4:**
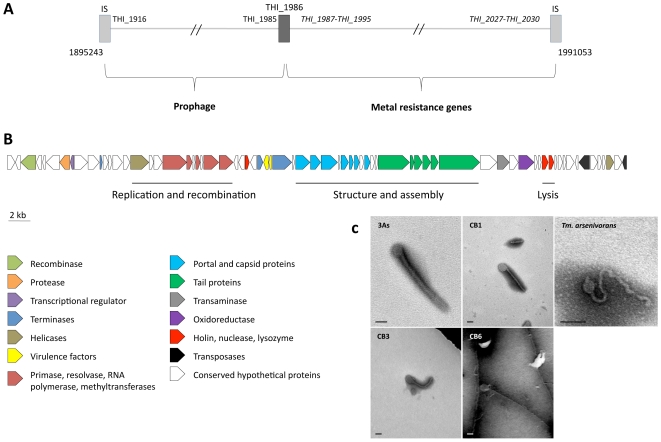
Prophage-like region and phage-like particle. (A) Schematic representation of the prophage-like region (55.6 kbp) and the contiguous metal resistance gene cluster (39.4 kbp). The total region is flanked by two ISs (light grey rectangles), and a partial transposase gene (THI_1986, dark grey rectangle) delimiting the two clusters. Genes in italics are duplicated in the *Thiomonas* sp. 3As genome; (B) organization of the prophage-like region. The 71 putative CDS, colored according to their category, are shown by arrows representing their direction. This cluster corresponds to the unique prophage-like region of the genome, containing notably structural, lysis and virulence associated phage genes; (C) transmission-electron micrographs of tailed bacteriophage-like particles from *Thiomonas* species. No bacteriophage-like particle was observed in *Thiomonas* sp. CB2 culture supernatant. Cultures were amended with mitomycin C (0.5 µg/mL). Bars: 100 nm. The 3As phage-like particle is hypothetically coded by the prophage-like region described above.

One GEI, ThGEI-J, contains a prophage region (55.6 kbp) and a cluster of 6 heavy metal resistance genes (39.4 kbp, i.e., *cad*, *cus*, *czc* and *sil* genes involved in resistance to Cd, Cu, Zn, Co and Ag) (Figure 4). The prophage region comprises 27 phage-related genes coding for structural and capsid or tail assembly proteins, replication, lysis and virulence factors. No conserved synteny with any previously described prophage could be observed. However, filamentous phage-like particles with icosahedral symmetry (capsid diameter of approximately 100 nm) and a various length tail (>600 nm), were observed by TEM from *Thiomonas* sp. 3As liquid cultures exposed to the phage lytic phase inducer mitomycin C. Similar phage-like particles were observed in growth culture supernatants from CB1, CB3, CB6 and *“Tm. arsenivorans”* (Figure 4) but not from CB2, Ynys1 and *Tm. perometabolis* (data not shown), in agreement with CGH results showing that the ThGEI-J is absent in these strains (Figure 3, Table S3, ArrayExpress database, accession number E-MEXP-2260). These observations suggest that this prophage-like region may be functional in 3As, CB1, CB3, CB6 and “*Tm. arsenivorans*” under stress conditions, resulting in the formation of phage-like particles.

### ThGEI-L and ThGEI-O Gene Content and Their Probable Acquisition by HGT

**Figure 5:**
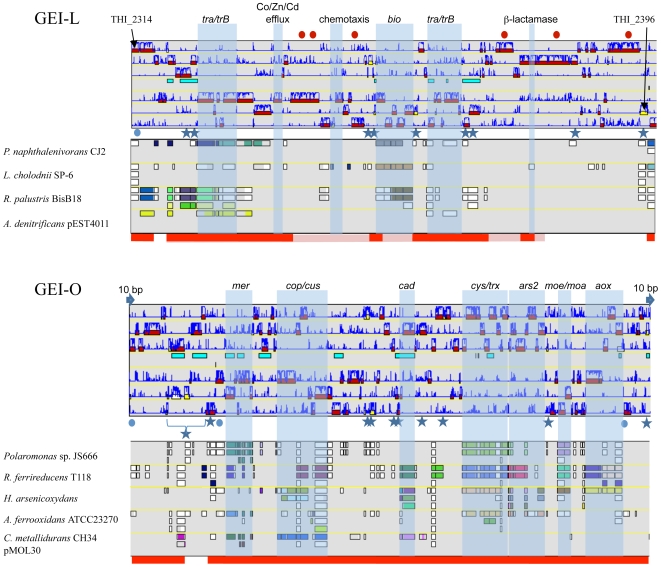
Genomic Island ThGEI-O containing genes involved in arsenic metabolism, and ThGEI-L. The best synteny results of the genes were obtained with genes of *Polaromonas naphthalenivorans* CJ2, *Leptothrix cholodnii* SP-6, *Rhodopseudomonas palustris* BisB18, *Achromobacter denitrificans* (ThGEI-L) or *Polaromonas* sp. JS666, *Rhodobacter ferrireducens*, *H. arsenicoxydans*, *Acidithiobacillus ferrooxidans* ATCC23270, and *Cupriavidus metallidurans* CH34 pMOL30 (ThGEI-O). Blue circles: genes originated from phage; Blue stars: transposases; Blue arrows: direct repeats. The genes conserved in 3As and CB strains or in AMD-originated strains (3As, CB strains and “*Tm. arsenivorans*”), are indicated by pink and red lines, respectively. Red dots indicate proteins with GGDEF and EAL domains.

GEIs contribute to the adaptation of microorganisms to their ecological niches and participate in genome plasticity and evolution [14]. Therefore, the environmental conditions may influence the loss or conservation of GEIs. Such hypothesis was checked by searching for genome similarities between strains originated from similar environments, i.e. AMD. To this aim, a hierarchical clustering was established based on genomic comparisons (Figure 2C). Interestingly, the clustering obtained was different from that of the 16S rRNA/*rpoA*-based phylogenetic trees (Figure 2A). Indeed, all strains that originated from AMD heavily loaded with arsenic, i.e. “*Tm. arsenivorans*” and strains 3As, CB1, CB2, CB3 and CB6, grouped together, whereas Ynys1 and *Tm. perometabolis* formed a distinct group. Genes possibly dispensable for AMD survival were therefore searched for and we identified 2541 CDS conserved in all strains originated from AMD, and these CDS may constitute the “AMD” core genome of *Thiomonas*. Interestingly, several genes present in the ThGEI-L and ThGEI-O were conserved in AMD-originated strains but absent in the other strains (Figure 5).

The ThGEI-L carries genes involved in panthotenate and biotine synthesis, and may confer auxotrophy to the strains carrying this island. Moreover, genes encoding Co/Zn/Cd efflux pump were present in this GEI. In addition, this island is particularly rich in proteins with GGDEF and EAL domains. The GGDEF or EAL domain proteins are involved in either synthesis or hydrolysis of bis-(3′-5′) cyclic dimeric GMP (c-di-GMP), an ubiquitous second messenger in the bacterial world that regulates cell-surface-associated traits and motility [43],[44]. Because of the presence of such genes in the vicinity of 2 genes involved in chemotaxis, this island may be important for *Thiomonas* strains to form biofilm, a cellular process involved in resistance to toxic compounds [37]. Indeed, some of these genes are duplicated in CB2 and CB3 and these two strains were shown to develop better biofilm synthesis capacities (Table S2). This island also carries several genes encoding integrases and components of T4SS, such as *virB1*, *virB4*, *trbBCD*, *traCEFGI*, *mob* and a *pilE*-like gene. The presence of such genes suggests that this island originated from an integrative and conjugative element (ICE) that disseminates via conjugation [45]. These observations suggest that this island may be still mobile.

ThGEI-O contains the *aox* and *ars2* genes (Figure 5). In addition, several other genes were found such as *mer*, *cop*, *cus* and *cad* genes involved in mercury, copper and cadmium resistance, respectively, *cys* involved in sulfate assimilation, and *moe/moa* genes involved in molybdenum cofactor biosynthesis as well as genes involved in exopolysaccharide production. The synteny of the genes found in *Thiomonas* sp. 3As ThGEI-O is not conserved in other arsenic-oxidizing bacteria (Figure 5). Several genes present in this region are duplicated in CB1 and CB6 (i.e. the *cop* and *aox* genes), or in CB3 (i. e. *mer*, *cop*, *cus*, *dsb*, *cys*, *ars*, *moe*/*moa*, *aox*, and *ptxB* genes). Only a single copy of this region is present in CB2 and 3As. PCR amplification and sequencing revealed that this ThGEI-O island is located in a different genomic region in CB2 as compared to 3As. Moreover, the *aox* and *ars* genes found in the ThGEI-O are duplicated in *“Tm. arsenivorans”* but absent in Ynys1 and *Tm. perometabolis*. Indeed, these two strains were unable to oxidize As(III), their As(III) resistance was lower than that of the other strains, and gene PCR amplification of *aox* and *ars2* failed with DNA extracted from these strains (Table S2). Altogether, the presence of at least one copy of these genes in all six strains isolated from arsenic-rich environments (i.e. 3As, CB strains and *“Tm. arsenivorans”*) suggests that this GEI is of particular importance for the growth of *Thiomonas* strains in their toxic natural environment, AMD.

The evolutionary origin of the ThGEI-L and –O was investigated using two different approaches. First, we performed the phylogenetic analysis of the 196 genes contained in these two islands (Table S4, Table S5). The resulting trees revealed that these genes have very different evolutionary histories suggesting that the formation of ThGEI-L and –O islands occurred through the recruitment of genes from various origins by HGT (Table S4, columns 2–5). Interestingly, the closest homologue of 30/75 and 22/121 3As genes, in ThGEI-L and –O respectively, is found in other *Thiomonas* species (mainly *Tm. intermedia*), suggesting that the formation of these islands occurred prior to the diversification of the *Thiomonas* genus and is thus relatively ancient. This hypothesis is supported by the global correspondence analysis (COA) performed on the entire genome. Our results did not reveal any particular codon usage bias, strengthening the hypothesis that these ThGEIs are ancient in *Thiomonas* genus (Figure S3). This may explained why the major genes of these two islands are present in 3As, CB1, CB2, CB3, CB6 and “*Thiomonas arsenivorans*”, as for example, the *ars2* operon and *aox* genes of the ThGEI-O. The phylogenetic analysis of *aox* genes revealed that all *Thiomonas* sequences grouped together with relationships that are very similar to organism relationships inferred with *rpoA* (Figure S4A and S4B). This indicates that these genes were already present in the *Thiomonas* ancestor and vertically transmitted in this genus, but lost in Ynys1 and *Tm perometabolis*. The phylogenetic analysis of the *arsB* genes, revealed that all *Thiomonas* sequences found in the ThGEI-O (i.e. *arsB2* from 3As, CB1, CB2, CB3, CB6 and “*Tm. arsenivorans*”), grouped together but not with *arsB1* genes that are part of the core genome of *Thiomonas*. Moreover, the evolutionary histories of these two proteins are different: ArsB1 proteins belong in a group containing mainly Alpha-Proteobacteria, whereas ArsB2 seems more closely related to Gamma-Proteobacteria (Figure S4C). These observations revealed that the *ars1* and *ars2* operons were not acquired from the same source or at the same time.

## Discussion

The exploration of the *Thiomonas* sp. 3As genome suggests that this strain has a wide range of metabolic capacities at its disposal. Many of them may make this bacterium particularly well suited to survive in its extreme environment, the acidic and arsenic-rich waters draining the Carnoulès mine tailings, as for example biofilm formation and heavy metal resistance. Moreover, some metabolic capacities are unique as compared to another arsenic-resistant bacterium, whose genome has been recently sequenced and annotated, *H. arsenicoxydans*, a strict chemoorganotroph, isolated from activated sludge [38]. The first metabolic idiosyncrasy of *Thiomonas* sp. 3As is its particular carbon and energy metabolic capacities. Indeed, several organic or inorganic electron donors, such as reduced inorganic sulfur compounds [31], could be used. Second, some *Thiomonas* strains, i.e. CB1, CB3, CB6 and *Tm. arsenivorans*, carry two copies of the *aox* operon. As far as could be ascertained, this is the first example of *aox* gene duplication. Finally, *Thiomonas* sp. 3As is able to grow at pH 3. Several genes potentially involved in acid resistance were found in *Thiomonas* genome. In addition, the Carnoulès toxic environment may cause severe DNA damage in *Thiomonas* sp. 3As, since arsenic is a co-mutagen that inhibits the DNA repair system [41]. DNA repair genes that have been previously shown to be induced in the presence of arsenic in *H. arsenicoxydans* were all found in *Thiomonas* sp. 3As genome, and the expression of *polA* has been shown to be induced in the presence of arsenic [8]. These observations suggest that this bacterium may respond to DNA damage. Nevertheless, we can hypothesize that these stressful conditions may lead to genomic rearrangements in *Thiomonas* genome. This could explain the important genomic diversity observed among the members of both the 3As species and the *Thiomonas* genus.

At the intra-species level, the dispensable genome defined by comparison of the CB strains with the 3As genome corresponds to 25.3% of *Thiomonas* sp. 3As genome. By comparison, this value is higher than that observed, with the same approach, in other bacteria such as *S. agalactiae* (18%) [46], lower than values calculated in the case of a pathogenic *E. coli* (32.4%) [47], and similar to the value obtained in *Bacillus subtilis* (27%) [48]. The value calculated for *Thiomonas* 3As and CB strains is very high, considering that these strains were isolated from the same site, closely related, and appear to share a recent common ancestor, as illustrated by our phylogenetical analyses. Consequently, we observed that despite strong sequence identities of housekeeping genes such as 16S rRNA or *rpoA*, the whole genome DNA-DNA hybridization value was relatively low, close to or less than 70%, for strains CB2 and CB3. Conventionally, this should indicate that these bacteria belong to separate evolutionary lineages and must be considered as different species [49]. However, the 16S rRNA-*rpoA* based analysis and CGH experiments revealed that the low DNA-DNA hybridization value correlates with the duplication or absence of several GEIs in these strains. Consequently, we proposed that despite low DNA-DNA hybridization values, these five strains do indeed belong to the same species. Similarly, the DNA-DNA hybridization values obtained with *Thiomonas* sp. 3As as compared to strains Ynys1 and *Tm*. *perometabolis* were very low, as previously observed [5],[10]. Altogether, the great genetic diversity observed in the present study by CGH experiments revealed that DNA-DNA hybridization method may not be appropriate to evaluate evolutionary lineages in *Thiomonas* strains. In this respect the CGH approach seems to be a reliable phylogenetic tool for typing these strains, as suggested in previous studies on other bacteria [47],[50].

19 GEIs constitute a large flexible pool of accessory genes that encode adaptive traits. Some of these genes are not required for survival in AMD, since they were not found in all AMD-originated strain genome and correspond therefore to the dispensable gene pool. On the other hand, CGH-based clustering analysis revealed a significant relationship between 3As, CB1-6 and *“Tm. arsenivorans”*, which originate from geographically distinct but similarly arsenic-rich environments. The *Thiomonas* sp. 3As strain and *“Tm. arsenivorans”* form two distinct groups on the basis of phylogenetical, physiological and genetic analyses. Nevertheless, the percent of flexible CDS of *Thiomonas* sp. 3As with *“Tm. arsenivorans”*, is relatively low (18.4%), as compared to the value obtained with Ynys1 and *Tm. perometabolis* (37.9% and 53.6%, respectively). This value obtained with *“Tm. arsenivorans”* was in the same order of magnitude as the value obtained with CB2 and CB3 (23% and 23.6%, respectively). Altogether, 70% of the *Thiomonas* sp. 3As genome was conserved among all strains originated from AMD. Interestingly, two GEIs were conserved or duplicated in all these strains originated from AMD, i.e. ThGEI-O that carries the arsenic-specific operons *ars2* and *aox*, and genes involved in heavy metal resistance, and ThGEI-L that carries several genes involved in heavy metal resistance, biofilm formation and/or motility. Therefore, these GEIs shared by these species are presumably part of the AMD-originated *Thiomonas* core genome. This observation suggests that the acquisition or loss of these GEIs contributes to the evolution of this subgroup of the *Thiomonas* genus and that the evolution of *Thiomonas* strains has been influenced by their similar environments.

Several observations suggest that *Thiomonas* genome evolved by acquiring GEIs through horizontal gene transfer or by genome rearrangement. In the case of two islands, ThGEI-L and –O, an in-deep phylogenetic analysis revealed that these islands have a composite structure probably due to secondary acquisition or losses/rearrangements of some genes. In the case of other GEIs, the existence of HGT is suggested by the fact that genes form syntenic blocks and their GC% were divergent from the rest of the genome. Three mechanisms, i. e. conjugation, transduction and natural transformation, known to be involved in HGT in bacteria [14] may explain GEIs acquisition in *Thiomonas*. One prophage was found in the *Thiomonas* genome and may contribute to horizontal gene transfer, as previously shown in pathogenic bacteria such as *Vibrio cholera*, *Yersinia pseudotuberculosis*, *Bartonella* [51]–[54], Cyanobacteria [55],[56] or for the transfer of pathogenic island from *S. aureus* to *Listeria monocytogenes* [57]. In addition, genes encoding Type IV secretion systems (T4SS) were carried by the pTHI plasmid and the ThGEI-L. It has been recently proposed that GEI-type T4SS are involved in the propagation of GEIs [45],[58]. Therefore, it could also be possible that *Thiomonas* acquired such islands by conjugation. Orthologs of *Neisseria gonorrhoeae* genes involved in natural transformation [59] were also found in *Thiomonas* sp. 3As genome, i.e. the *pil* genes encoding a type IV pili components and *comALMP*. This suggests that *Thiomonas* strains are able to acquire exogenous DNA. Finally, several observations suggest that *Thiomonas* genome has undergone genomic rearrangements contributing to its evolution, as illustrated for the two GEIs, ThGEI-L and -O. Such rearrangements may be promoted by repeat sequences or duplications, that are at the origin of recombination [60]. Indeed, repeats sequences represent 7.62% of the *Thiomonas* sp. 3As genome, and some of the loci found in the GEIs are duplicated with high sequence identities, as ThGEI-C and ThGEI-S that are almost identical. In addition, several IS elements are highly similar, sharing more than 70% nucleotide identity. Interestingly, the majority of the ISs present in the *Thiomonas* sp. 3As genome are found in GEIs. This observation suggests that ISs duplication has played a significant role in both assembly and evolution of these islands, or participated in GEI reshuffling. Altogether, conjugation, transduction, natural transformation and recombination may be at the origin of the high genomic content divergence observed among *Thiomonas* strains.

In conclusion, evidences presented here suggest that *Thiomonas* sp. 3As has acquired some of its particular capacities that contribute to its survival and proliferation in AMD by horizontal gene transfer and genomic rearrangement. Furthermore, these data revealed a high degree of genetic variability within the *Thiomonas* genus, even at the intra-species level. Indeed, the analysis of duplications and deletions of GEIs in several strains revealed the huge significance of these GEIs in the evolution of the *Thiomonas* genus, as well as the influence of the natural environment on the genomic evolution of this extremophile. The majority of intra or inter-species comparisons carried out thus far have concerned pathogens. Our analysis shows that GEIs play also an important role in the evolution of environmental isolates exposed to toxic elements.

## Materials and Methods

### Bacterial Strains

*Thiomonas* sp. 3As was obtained from the acidic waters draining the Carnoulès mine tailings, southeastern France [5]. *Thiomonas* strains CB1, CB2, CB3 and CB6 were isolated from the same site: briefly, the isolates were purified by repeated single colony isolation on either R2A medium (Difco; strains CB1, CB2 and CB3) or 100∶10 medium ([61]; strain CB6). Physiological, phylogenetic and genetic analyses of these four strains were performed as described previously (Tables S2, S6, [8]). Strains *Thiomonas* Ynys1 [62], *Tm. perometabolis* [63] and “*Tm. arsenivorans*” [9] were cultivated as previously described [8]. DNA-DNA hybridization was carried out as described by [64] under consideration of the modifications described by [65] using a model Cary 100 Bio UV/VIS-spectrophotometer equipped with a peltier-thermostated 6×6 multicell changer and a temperature controller with *in situ* temperature probe (Varian).

### DNA Preparation, Sequencing, and Annotation

DNA was extracted and purified from liquid cultures of pure isolates as previously described [8]. The complete genome sequence of *Thiomonas* sp. 3As was determined using the whole-genome shotgun method. Three libraries were constructed, two plasmids and one BAC to order contigs, as previously described [38]. From these libraries, 26,112, 7,680 and 3,840 clones were end-sequenced, and the assembly was performed with the Phred/Phrap/Consed software package (www.phrap.com), as described previously [38]. An addition of 3,292 sequences was needed for the finishing phase. Coding sequences were predicted as previously described [38]. Putative orthology relationships between two genomes were defined by gene pairs satisfying either the Bidirectional Best Hit criterion or an alignment threshold (at least 40% sequence identity over at least 80% of the length of the smallest protein). These relationships were subsequently used to search for conserved gene clusters (synteny groups) among several bacterial genomes using an algorithm based on an exact graph-theoretical approach [66]. This method allowed for multiple correspondences between genes, detection of paralogy relationships, gene fusions, and chromosomal rearrangements (inversion, insertion/deletion). The ‘gap’ parameter, representing the maximum number of consecutive genes that are not involved in a synteny group, was set to five. Manual validation of automatic annotations was performed in a relational database (ArsenoScope, https://www.genoscope.cns.fr/agc/mage/wwwpkgdb/MageHome/index.php?webpage=mage) using the MaGe web interface [67]. The EMBL (http://www.ebi.ac.uk/embl) accession numbers for the genome of *Thiomonas* sp. 3As are FP475956 (chromosome) and FP475957 (plasmid).

### Comparative Genome Hybridization (CGH) Array

A custom 385K array for the *Thiomonas* sp. 3As chromosome and plasmid was designed and constructed by NimbleGen Systems. This DNA array encompasses 3,645 CDS of the 3As genome. Probe length was 50 nt and current mean probe spacing was 7 nt. Genomic DNAs from all strains were extracted with the Wizard Genomic DNA Purification Kit (Promega). DNA samples were labeled and purified using the BioPrime Array CGH Genomic Labeling System protocol (Invitrogen). Test (Cy3-labeled) and reference (*Thiomonas* sp. 3As genomic DNA, Cy5-labeled) genomic DNAs were combined (400 pmol fluorescent dye each) and were co-hybridized to the array for 16 h at 42°C in a MAUI Hybridization System (BioMicro System) and slides were washed according to NimbleGen's recommendations. Dye swap experiments were used to compare *Thiomonas* sp. 3As and *Thiomonas* sp. CB1 genomic DNAs. Arrays were scanned with an Axon 4000B scanner. Data were acquired and analyzed using NimbleScan 2.0 and SignalMap 1.9 software (Roche, NimbleGen) and analyzed using the Partek Genomics Suite software (Partek Incorporated, St. Louis, Missouri, U.S.A.). Briefly, log2-ratios (Cy5/Cy3) were calculated using the segMNT algorithm and gains and losses of genomic material were identified using Partek Genomics Suite Software, as follows: the files were imported and normalized with the qspline normalization [68] by NimbleScan. These data were then imported into the Partek Genomics Suite Soft. The segmentation was performed using the circular binary segmentation algorithm from Olshen *et al*. [69]. Permutations are used to provide the reference distribution to check a second time. 1000 permutations are run using the Partek software. If the resulting p-value is below the threshold (p = 0.01), then a breakpoint is added. To verify deletions, PCRs were performed as described in supplementary materials, using primers designed to anneal at the borders of the expected deletions. The CGH data are available in the database ArrayExpress, with the accession number E-MEXP-2260.

### Phage Excision and Electron Microscopy

Phage formation was induced by treating exponential cultures with mitomycin C (0.5 µg/mL) for 24 h. The suspension was negatively stained with 16% ammonium molybdate for 10 seconds and dried over Formvar*-*coated nickel grids. Grids were examined at 40,000-fold magnification using a Hitachi 600 transmission electron microscope at 75 kV and photographed using a Hamamatsu ORCA*-*HR camera (Hamamatsu City, Shizuoka, Japan) with the AMT software (Advanced Microscopy Techniques Corp., Danvers, MA).

### Phylogenetic and Correspondence Analysis

For each CDS, homologues were searched in NCBI databases. The 300 sequences with the best score were aligned using ClustalW [70]. Alignments were checked by hand and positions with more than 5% of gaps were automatically removed. Neighbor-Joining trees were constructed and analyzed to determine the evolutionary origin of each CDS (Table S4). The correspondence analysis (COA) [71] was performed using the library FactoMineR (http://factominer.free.fr) from the statistical package R (http://www.r-project.org). For all annotated genes of *Thiomonas* sp. 3As, we determined all the relative synonymous codon usage values [72] obtaining a matrix where the rows represent the genes and the 57 columns are the RSCU values for individual codons. As usual, the 3 TER codons were excluded from the analysis. Codons corresponding to Cystein (TGC/TGT) and the duet of Arginine (AGG/AGA) were also removed from the analysis as they induce systematic artefactual biases [73].

## Supporting Information

Figure S1 Circular representations of *Thiomonas* chromosome and pTHI plasmid. Gene organization found in (A) the *Thiomonas* sp. 3As chromosome. The localisation of the 19 GEIs (A-S) is schematized with grey triangles; (B) the plasmid pTHI. Circles display (from the outside): (1) GC percent deviation (GC window - mean GC) in a 1000-bp window; (2) Predicted CDSs transcribed in the clockwise direction (red); (3) Predicted CDSs transcribed in the counterclockwise direction (blue); (4) GC skew (G+C/G−C) in a 1000-bp window; (5) Transposable elements (pink) and pseudogenes (grey).
(26.67 MB TIF)

Figure S2 Duplication of genes found in the several GEIs. Only those with high amino acid identities are shown.
(0.95 MB TIF)

Figure S3 Factor maps obtained by crossing the first and second axes of the correspondence analysis computed on 3,632 *Thiomonas* sp. 3As genes. For clarity, genes that are not harbored in the 19 GEIs (defined in Table S3) are not represented.
(0.94 MB TIF)

Figure S4 Phylogenetic trees of arsenic specific genes compared to *rpoA*. blue: gamma-Proteobacteria; brown: Hydrogenophilales; orange: Methylophilales; light green: Nitrosomonadales; deep green: Rhodocyclales; red: Neisseriales; pink: Burkholderiales. (A) *rpoA*; (B) *aoxB*; (C) *arsB*.
(2.19 MB TIF)

Table S1 List of ISs present in *Thiomonas* sp. 3As genome and plasmid.
(0.05 MB XLS)

Table S2 Summary of physiological and genetic data obtained from the *Thiomonas* strains used in this study.
(0.04 MB DOC)

Table S3 Genomic Islands (GEIs) or islets found in the *Thiomonas* sp. 3As genome.
(0.04 MB XLS)

Table S4 Phylogenetic analysis of the 75 genes contained in the ThGEI-L island.
(0.04 MB XLS)

Table S5 Phylogenetic analysis of the 121 genes contained in the ThGEI-0 island.
(0.05 MB XLS)

Table S6 PCR targets and GenBank Accession IDs of strains used in this study.
(0.03 MB DOC)
