# Supplementary figures and images for: Structure, Function, and Evolution of the Thiomonas spp. Genome
Source: PLoS Genet. 2010 Feb 26;6(2):e1000859. doi: 10.1371/journal.pgen.1000859 (PMC2829063; doi:10.1371/journal.pgen.1000859)

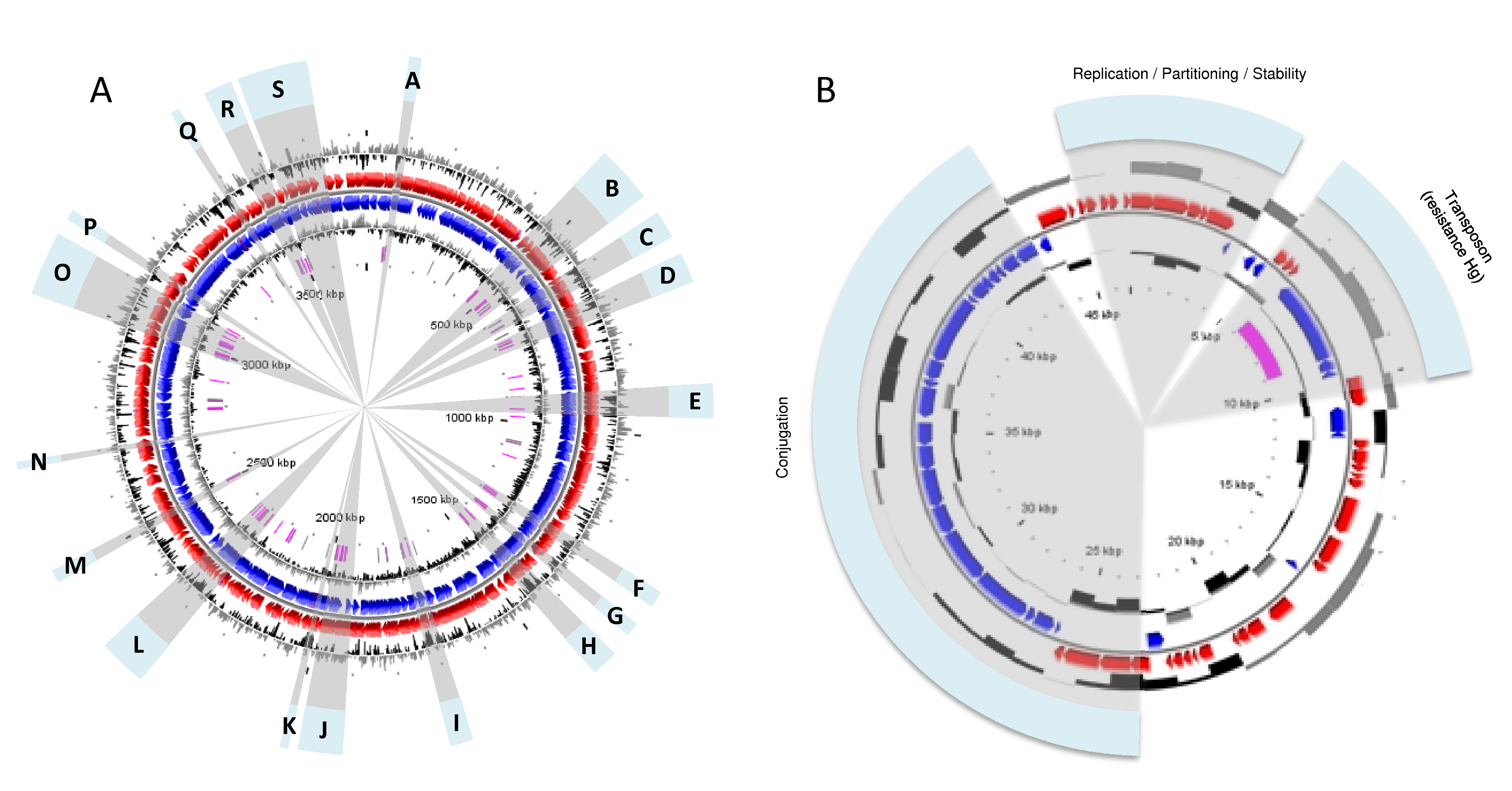

Supplement: Figure S1 — Circular representations of Thiomonas chromosome and pTHI plasmid. Gene organization found in (A) the Thiomonas sp. 3As chromosome. The localisation of the 19 GEIs (A-S) is schematized with grey triangles; (B) the plasmid pTHI. Circles display (from the outside): (1) GC percent deviation (GC window - mean GC) in a 1000-bp window; (2) Predicted CDSs transcribed in the clockwise direction (red); (3) Predicted CDSs transcribed in the counterclockwise direction (blue); (4) GC skew (G+C/G−C) in a 1000-bp window; (5) Transposable elements (pink) and pseudogenes (grey). (26.67 MB TIF) [file pgen.1000859.s001.tif]

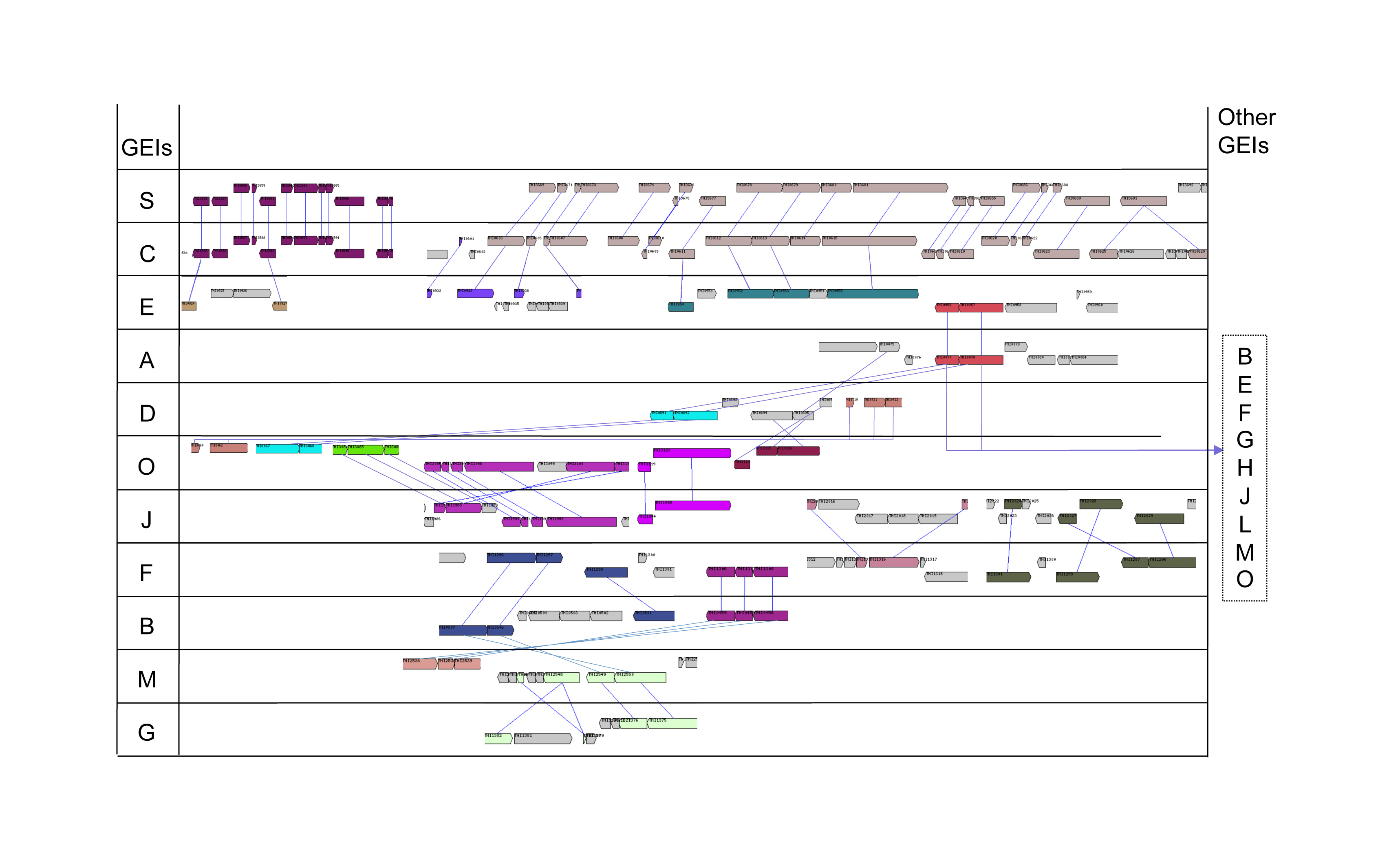

Supplement: Figure S2 — Duplication of genes found in the several GEIs. Only those with high amino acid identities are shown. (0.95 MB TIF) [file pgen.1000859.s002.tif]

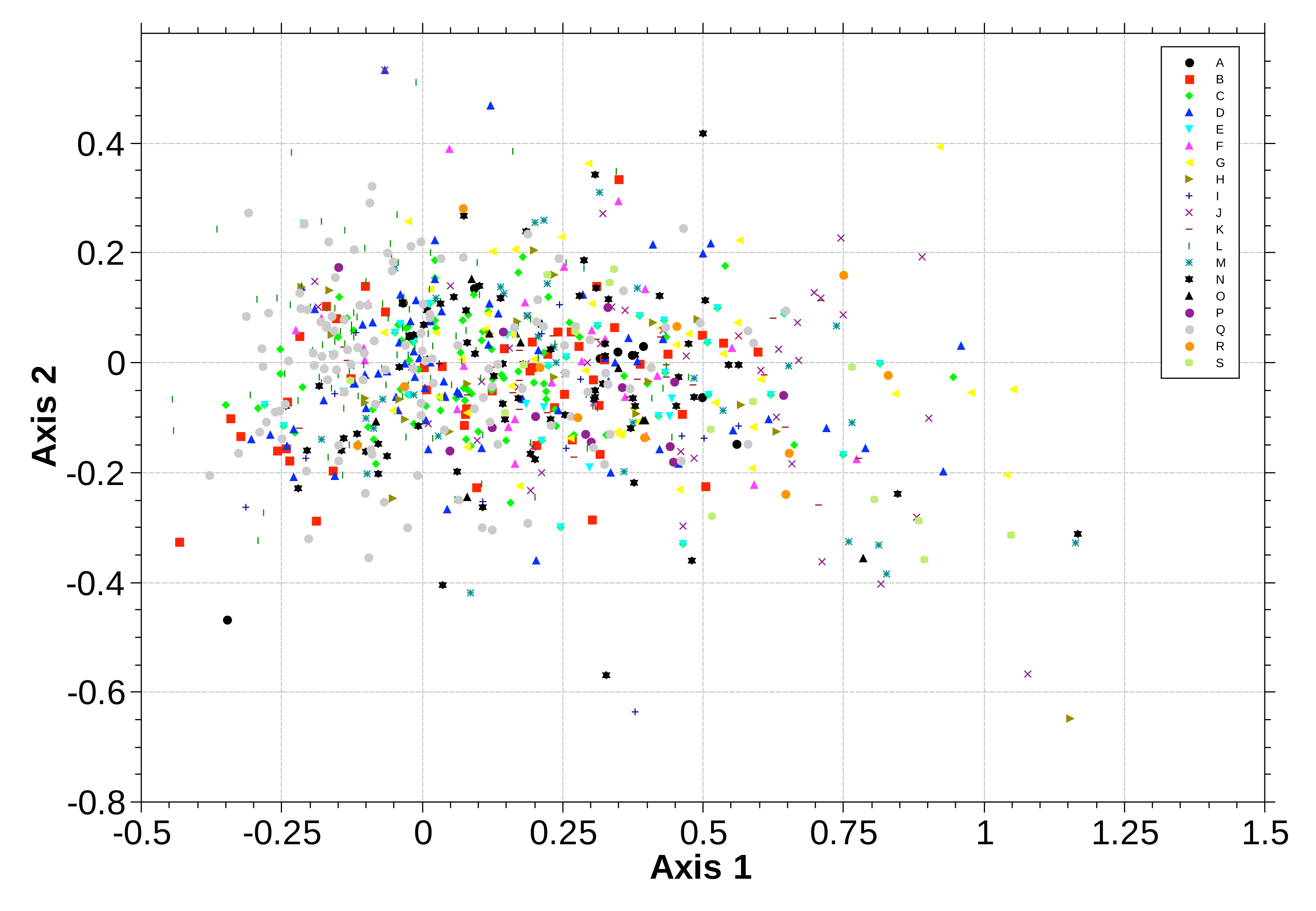

Supplement: Figure S3 — Factor maps obtained by crossing the first and second axes of the correspondence analysis computed on 3,632 Thiomonas sp. 3As genes. For clarity, genes that are not harbored in the 19 GEIs (defined in Table S3) are not represented. (0.94 MB TIF) [file pgen.1000859.s003.tif]

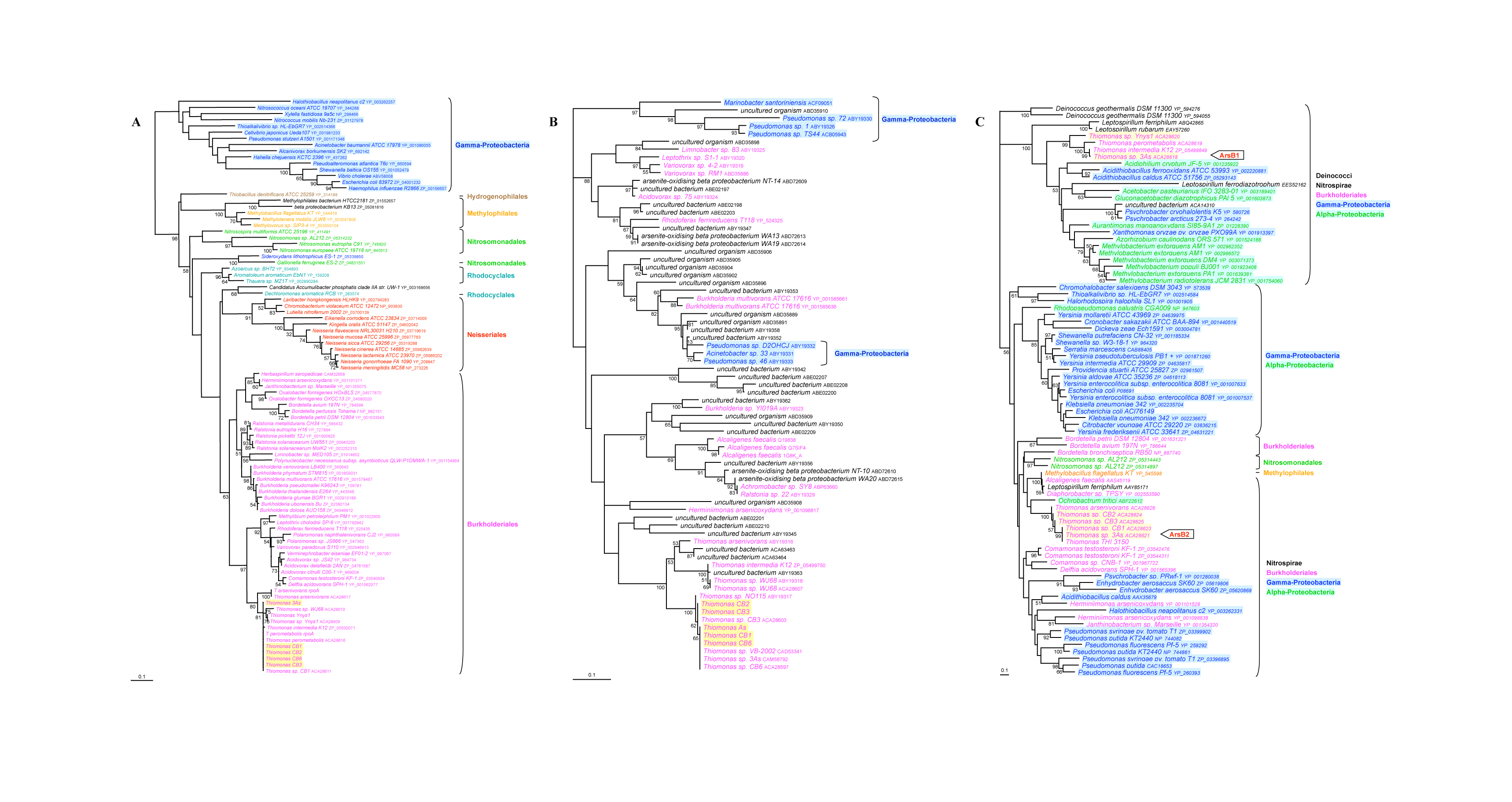

Supplement: Figure S4 — Phylogenetic trees of arsenic specific genes compared to rpoA. blue: gamma-Proteobacteria; brown: Hydrogenophilales; orange: Methylophilales; light green: Nitrosomonadales; deep green: Rhodocyclales; red: Neisseriales; pink: Burkholderiales. (A) rpoA; (B) aoxB; (C) arsB. (2.19 MB TIF) [file pgen.1000859.s004.tif]
