# Supplementary material for: Structure, Function, and Evolution of the Thiomonas spp. Genome
Source: PLoS Genet. 2010 Feb 26;6(2):e1000859. doi: 10.1371/journal.pgen.1000859 (PMC2829063; doi:10.1371/journal.pgen.1000859)
Supplement: Table S2 — Summary of physiological and genetic data obtained from the Thiomonas strains used in this study. (0.04 MB DOC) [file pgen.1000859.s006.doc]

|  | | | | | | | | | | | | | | | | | |
| --- | --- | --- | --- | --- | --- | --- | --- | --- | --- | --- | --- | --- | --- | --- | --- | --- | --- |
| *Thiomonas strains* | Oxidation As(III) | MIC (mM) | | | Arsenic-related gene PCR amplification | | | | Motility^a^ | | Effect of arsenite on strains motility^b^ | | Biofilm formation ^c^ | | Reference | |  |
|  |  | | As(III) | As(V) | | *aoxAB* | *arsB1* | *arsB2* | |  | |  | |  | |  | |
|  |  |  |  |  |  |  |  |  |  |  |  |  |  |  |  |  |  |
| 3As | + | | >6.0 | >50 | | + | + | + | | 2.9 | | 0 | | 0.19 ± 0.045 | | This study and [5] | |
| CB1 | + | | >6.0 | >50 | | + | + | + | | 9.1 | | + (16%) | | 0.15 ± 0.039 | | This study | |
| CB2 | + | | >6.0 | >100 | | + | + | + | | 0 | | nd | | 0.31 ± 0.015 | | This study | |
| CB3 | + | | >6.0 | >50 | | + | + | + | | 0 | | nd | | 0.40 ± 0.094 | | This study | |
| CB6 | + | | >6.0 | >6 | | + | + | + | | 3.1 | | + (18%) | | 0.11 ± 0.032 | | This study | |
| Ynys1 | - | | >2.7 | >6 | | - | + | - | | 5.6 | | - (35%) | | nd | | [9] | |
| *Tm. perometabolis* | - | | >2.7 | >100 | | - | + | - | | 0 | | nd | | nd | | [9] | |
| *Tm. arsenivorans* | + | | >6.7 | >50 | | + | - | + | | 4.5 | | + (24%) | | nd | | [9] | |
| ^a^ Diameter (mm) of swarming ring formed on 0.3% agar plates after 72h incubation expressed as a difference with non motile strains (forming colonies of <3 mm diameter);  ^b^ Motility was tested in the presence of 1.33 mM As(III). “+” indicates a diameter of swarming ring higher than in absence of As(III), “-” a lower one and “0” no difference;  ^c^ Measured by cristal violet staining after 72 h incubation with 1.33 mM As(III); nd: not determined | | | | | | | | | | | | | | | | | |
