# Supplementary material for: Structure, Function, and Evolution of the Thiomonas spp. Genome
Source: PLoS Genet. 2010 Feb 26;6(2):e1000859. doi: 10.1371/journal.pgen.1000859 (PMC2829063; doi:10.1371/journal.pgen.1000859)
Supplement: Table S6 — PCR targets and GenBank Accession IDs of strains used in this study. (0.03 MB DOC) [file pgen.1000859.s010.doc]

| Strain | 16S | *rpoA* | *aoxAB* | *arsB1* | *arsB2* |
| --- | --- | --- | --- | --- | --- |
| 3As | AM492684^ab^ | EU339226^b^ | EU339209 | EU339214 | EU339217 |
| CB1 | FJ014921^b^ | EU339225^b^ | EU339210 | n/s | EU339219 |
| CB2 | FJ014922^b^ | EU339229 | EU339212 | n/s | EU339220 |
| CB3 | FJ014924^b^ | EU339227^b^ | EU339211 | n/s | EU339221 |
| CB6 | FJ014923^b^ | EU339228^b^ | EU339208 | n/s | EU339218 |
| ^a^ Accession IDs from other studies [5]; ^b^ Sequences share 100% nucleotide identity; n/s, sequences not submitted: the *arsB1* and *arsB2* sequences obtained with the internal primers were short and therefore were not submitted to the GenBank sequence depository (see [9]) | | | | | |
